# Dichotomous outcomes vs. survival regression models for identification of predictors of mortality among patients with severe acute respiratory illness during COVID-19 pandemics

**DOI:** 10.3389/fpubh.2023.1271177

**Published:** 2023-12-06

**Authors:** Karen Ingrid Tasca, Camila Gonçalves Alves, Rejane Maria Tommasini Grotto, Leonardo Nazario de Moraes, Patrícia Akemi Assato, Carlos Magno Castelo Branco Fortaleza

**Affiliations:** ^1^Department of Infectious Diseases, Botucatu Medical School (FMB), São Paulo State University (Unesp), Botucatu, São Paulo, Brazil; ^2^Department of Biotechnology and Bioprocess, School of Agriculture (FCA), São Paulo State University (Unesp), Botucatu, São Paulo, Brazil; ^3^Clinical Hospital of Botucatu Medical School (HCFMB), Botucatu, Brazil

**Keywords:** SARI, COVID-19, multivariable models, Poisson regression, Cox regression, clinical predictors

## Abstract

**Introduction:**

As the studies predicting mortality in severe acute respiratory illness (SARI) have inferred associations either from dichotomous outcomes or from time-event models, we identified some clinical-epidemiological characteristics and predictors of mortality by comparing and discussing two multivariate models.

**Methods:**

To identify factors associated with death among all SARI hospitalizations occurred in Botucatu (Brazil)/regardless of the infectious agent, and among the COVID-19 subgroup, from March 2020 to 2022, we used a multivariate Poisson regression model with binomial outcomes and Cox proportional hazards (time-event). The performance metrics of both models were also analyzed.

**Results:**

A total of 3,995 hospitalized subjects were included, of whom 1338 (33%) tested positive for SARS-CoV-2. We identified 866 deaths, of which 371 (43%) were due to the COVID-19. In the total number of SARI cases, using both Poisson and Cox models, the predictors of mortality were the presence of neurological diseases, immunosuppression, obesity, older age, and need for invasive ventilation support. However, the Poisson test also revealed that admission to an intensive care unit and the COVID-19 diagnosis were predictors of mortality, with the female gender having a protective effect against death. Likewise, Poisson proved to be more sensitive and specific, and indeed the most suitable model for analyzing risk factors for death in patients with SARI/COVID-19.

**Conclusion:**

Given these results and the acute course of SARI and COVID-19, to compare the associations and their different meanings is essential and, therefore, models with dichotomous outcomes are more appropriate than time-to-event/survival approaches.

## Introduction

SARS-CoV-2 was the most common–but not the only-agent of Severe Acute Respiratory Illness (SARI) during the COVID-19 pandemic, and this syndrome is responsible for a significant number of hospital admissions and is the major cause of death and morbidity in low- and middle-income countries. Before COVID-19, etiologic agents were often undetermined due to the lack of molecular diagnostics in hospitals and clinics. Studies have focused on the impact of SARS-CoV-2 and other co-circulating viruses on the mortality of patients admitted for SARI, as well as in other predictors of unfavorable outcomes (1–4). This studies have emerged at a frantic pace resulting from the urgency of responses regarding disease treatment and prevention, making it difficult to interpret this abundance of results, mainly because there is a diversity of statistical methods applied for the same purpose, given this particular disease and its outcome.

Despite vaccination, COVID-19 is still killing many people worldwide. Thus, establishing mortality predictors is the key to taking steps to slow down this scenario. For this, the behavior of different variables correlated with the COVID-19 prediction as sex, age, ethnicity and socio-economic backgrounds must be considered facts that require improved mortality models (5). To use prediction models within clinical practice guidelines to make decisions is still necessary for patient care (6, 7). Considering that estimates of probabilities are rarely based on a single predictor, it is inherently multivariable. Therefore, prediction models are tools that combine multiple predictors by assigning relative weights to each predictor to obtain a risk or probability (6).

Studies predicting mortality in SARI and/or COVID-19 have inferred associations either from dichotomous outcomes or time-event models. Although these associations seem similar, they have different meanings. For example, given that cumulative outcome at a particular point of time is simpler and can be analyzed with logistic regression, a type of multivariable analysis that is relatively easier to conduct and interpret than proportional hazards analysis, there are so many published papers using time to outcome. It is probably because clinical medicine consists more of treatment than cure or because the Cox model allows the incorporation of subjects with differing lengths of follow-up in its analysis, a common choice in longitudinal studies (8). However, probabilistic mortality models usually assume that deaths are independent, identically distributed Yes/No events. In this case, it was estimated by fitting Poisson distributions to mortality counts with known exposures, using a log link function, an improvement to standard continuous mortality models (5).

Here we proposed to verify clinical-epidemiological characteristics and to identify mortality predictors in SARI inpatients, applying multivariate models of dichotomous (Poisson) and time-event (Cox) outcomes in a city that promoted a mass vaccination campaign against COVID-19 in its population, to compare if factor associated with death and respective risk measurements remained similar using different multivariable analysis methods.

## Materials and methods

### Data source

The case definition for SARI according to Brazilian surveillance is: an individual with ^*^Syndrome Influenza presenting dyspnea/respiratory distress, or persistent chest pressure, or O2 saturation lower than 95% on room air, or bluish coloration of lips or face. (^*^SG: individual with an acute respiratory condition characterized by at least two of the following signs and symptoms: fever - even if referred -, chills, sore throat, headache, cough, runny nose, smell or taste disturbances). For the purpose of notification in SIVEP-Gripe, hospitalized cases of SARS or deaths from SARS regardless of hospitalization should be considered (9).

Data on SARI hospitalization and death were collected from the Brazilian hospitalization database operated by the Ministry of Health-the Influenza Surveillance System Data Repository (SIVEP-Gripe), which monitors SARI hospitalization cases in Brazil. Thus, the epidemiological surveillance of Botucatu provided us the report of its citizens hospitalized for SARI on 11 April 2022.

Botucatu is a city in inner São Paulo State, with an estimated population of 142,092 (10). The hospital admissions occurred in private and public hospitals (through the health program within Brazil's socialized Unified Health System–SUS), including the Clinical Hospital, a university hospital that provides tertiary care for this city and surrounding municipalities.

All inpatients were obtained from the SIVEP-Gripe database from the 1st of March 2020 to the 31st of March 2022, including residents of Botucatu. After acquiring the database, the data was treated (excluding people with missing “final evolution/outcome” and “final classification” fields), and the variables were binary transformed. All vaccines were manually added to our database by consulting the State of São Paulo's Vaccination Recording System against COVID-19 (VaciVida). Sensitive data were subsequently anonymized to proceed with the statistical analyses.

### Study design, variables, and groups

We performed a cohort retrospective analytical study with secondary data from SIVEP-Gripe.

The main outcome consisted of cure/recovery (discharge) and deaths. We considered the following predictors to identify risk factors associated with the occurrence of death: age, sex (male and female), and the presence or absence of pre-existing comorbidities (postpartum or pregnancy, cardiovascular, renal, neurological, hematological, or hepatic comorbidities, diabetes, chronic respiratory disorder, obesity, Down syndrome or immunosuppression). Clinical course was reported in terms of the need (or not) for non-invasive (NIVS) or invasive ventilation support (IVS) and the admission (or not) to an intensive care unit (ICU). For vaccination data, were included the number of doses received (none, one, two, three, or four) at the time of the SIVEP-gripe notification.

Variables regarding signs and symptoms present on admission (fever, cough, sore throat, shortness of breath, respiratory distress, gastrointestinal symptoms, and oxygen saturation) were included to the descriptive analysis. Besides, comorbidity data were also categorized according to the number of pre-existing conditions (none, one, and ≥two/multimorbidity).

The first group analyzed was all SARI cases-for all hospitalization notifications contained in SIVEP-gripe that remained after applying the exclusion criteria. Then, a filter was used in the database to analyze only people with a confirmed diagnosis of SARS-CoV-2, here referred as the “COVID-19 subgroup.” It is important to emphasize that all patients were tested for SARS-CoV-2.

### Statistical analysis

We calculated descriptive statistics for inpatient characteristics. Quantitative variables were expressed as mean ± standard deviation (SD) values, and categorical variables were expressed as absolute (*n*) and relative (%) frequencies values. Chi square test was used for testing relationships between categorical variables.

Poisson regression with binomial outcome estimated the crude and adjusted for the confounders variables in order to obtain relative risks (RR) and 95% confidence intervals (95% CI). The Cox proportional-hazards (time-event) model was used to estimate the death risk by the studied variables, being these associations expressed as hazard ratios (HR) with 95% CI and considering the time from hospital admission to outcome. A single-step model included demographic data, comorbidities, care needs, and vaccines. A two-sided *p*-value ≤ 0.05 was considered as statistical significance.

Besides, considering the predicted values obtained by each models, a receiver operating characteristic curve (ROC) curve was calculated in order to compare the area under the curve (AUC), specificity and sensitivity values (for death outcome).

Analyses were performed both for all SARI cases and those confirmed for COVID-19 singly, using SAS for Windows (version 9.4) software.

### Ethical issues

The local Research Ethics Committee of Botucatu Medical School (FMB/Unesp) approved the study (CAAE: 57919122.9.0000.5411) without the need for informed consent.

Additionally, this study complied with the Resolution 466/2012 and 510/2016 of the Brazilian National Health Council and with the Strengthening the Reporting of Observational Studies in Epidemiology (STROBE) guidelines (Supplementary material 1).

## Results

**Figure 1:**
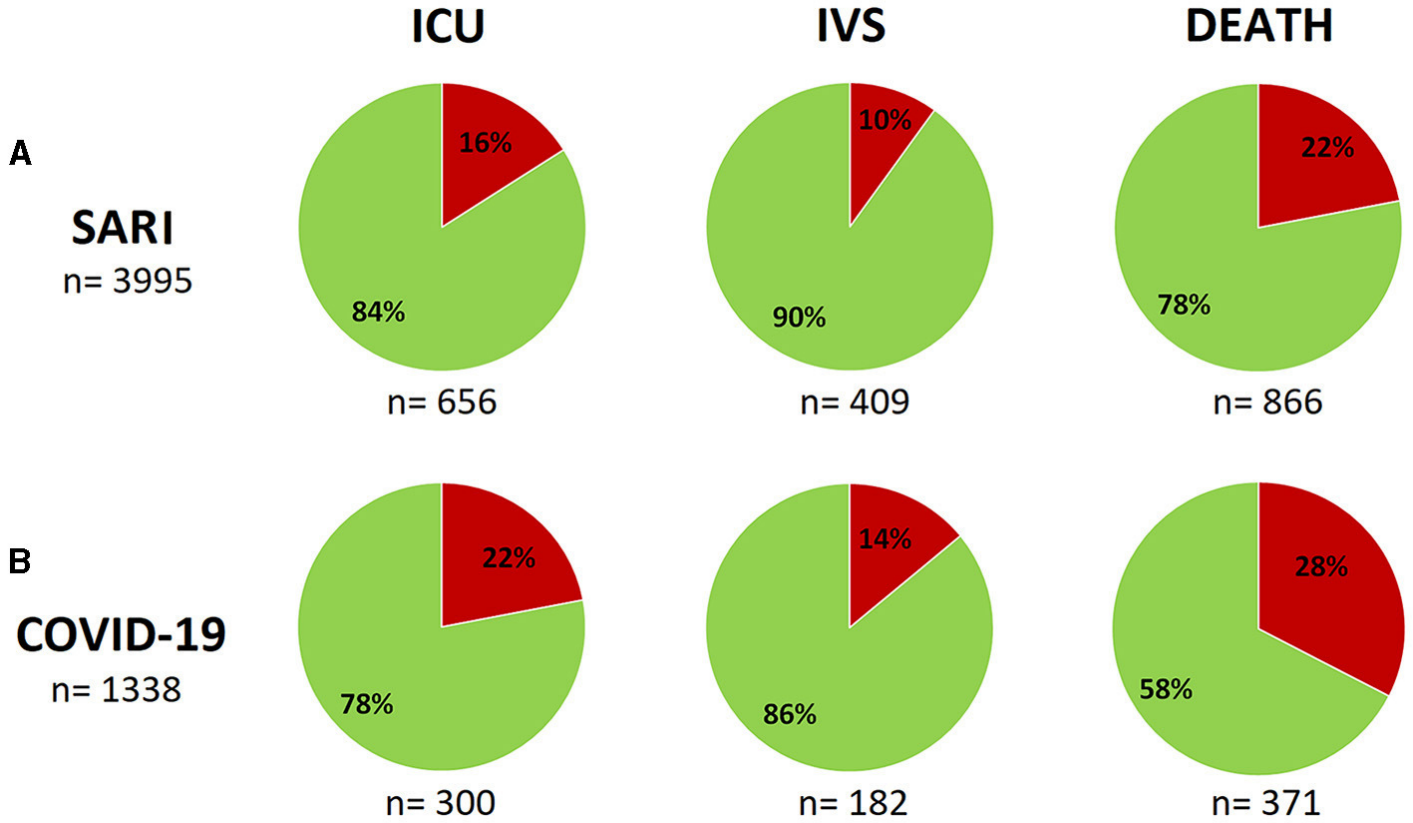
Intensive care unit (ICU) admissions, invasive ventilation support (IVS) and deaths in patients residing in Botucatu/SP, hospitalized for SARI **(A)** and specifically the COVID-19 subgroup **(B)** during the period from March 2020 to March 2022. The frequencies are shown by different colors - in red: “yes” (which shows the “number of sample” right below the corresponding graph); green: “no.” Chi square test (difference in proportions) showed differences for all variables/outcomes when SARI group was compared to COVID-19 subgroup (*p* < 0.001). ICU comparison (**A** vs. **B**): *p* < 0.0001; IVS: *p* = 0.0009; Death: *p* < 0.0001.

Out of the 3,995 hospitalized people included, 1,338 (33%) tested positive for SARS-CoV-2. Among all those hospitalized, 656 (16%) required ICU admission, and 409 (10%) required IVS. These numbers were 300 (22%) and 182 (14%), respectively, for COVID-19 patients. In total, 866 (22%) deaths were identified, 371 (28%) were in the COVID-19 group. Therefore, hospitalization for COVID-19 was more severe than other respiratory viruses (*p* < 0.001), and the in-hospital mortality by COVID-19 was 28%. Figure 1 shows these results. Therefore, the cumulative COVID-19 mortality rate in Botucatu was 3%.

**Figure 2:**
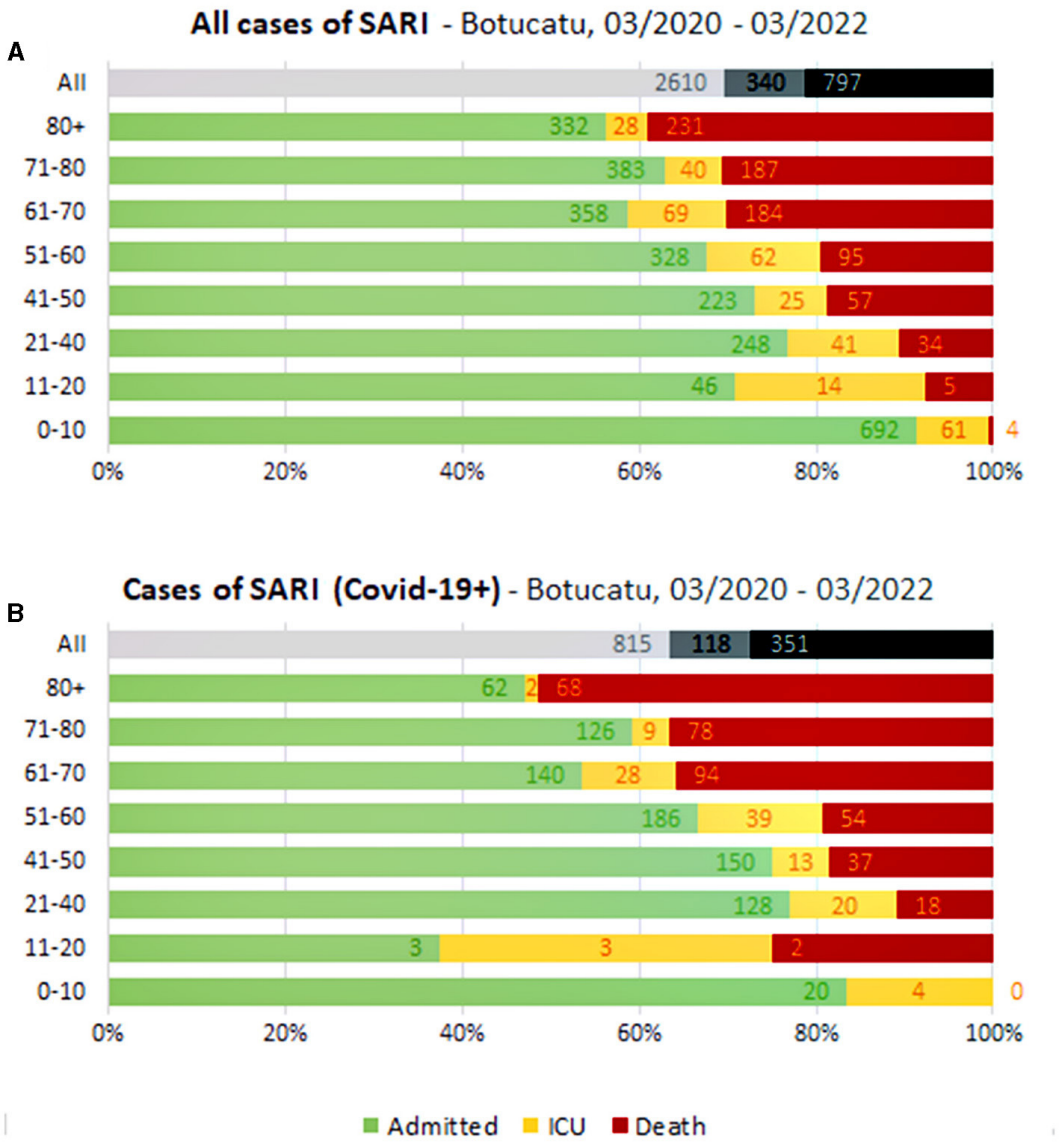
All cases of SARI **(A)** and cases of SARI by COVID-19 **(B)** from Botucatu/SP: hospitalizations, intensive care unit (ICU) admissions, and deaths by age groups during 2 years.

The average age of the patients was 52 (±25) years old in the SARI group and 59 (±18) in the COVID-19 subgroup. In general, we observed that almost 50% of all SARI hospitalization (and the same proportion to the COVID-19 subgroup) occurred in older people (aged 60+). For all deaths reported, 46% were older people for SARI and 70% for the COVID-19 subgroup (Figure 2).

The proportion of male patients ranged from 52% for SARI to 55% for COVID-19 subgroup. The most common clinical manifestations among all hospitalized, present in at least 50% of them, were low oxygen saturation (SpO2 < 95%), dyspnea, coughing, respiratory decompensation, and fever (data not showed).

Most of the individuals included in the study were unvaccinated against COVID-19; specifically in the COVID-19 subgroup, 68% (*n* = 912) were unvaccinated, and the vaccinated rates considering different doses received were 15% (dose 1), 12% (dose 2), 5% (dose 3) and 0.2% (dose 4). Among those vaccinated with 1 or 2 doses, most had received AstraZeneca (*n* = 192) or Coronavac (*n* = 164) (Supplementary material 2).

**Figure 3:**
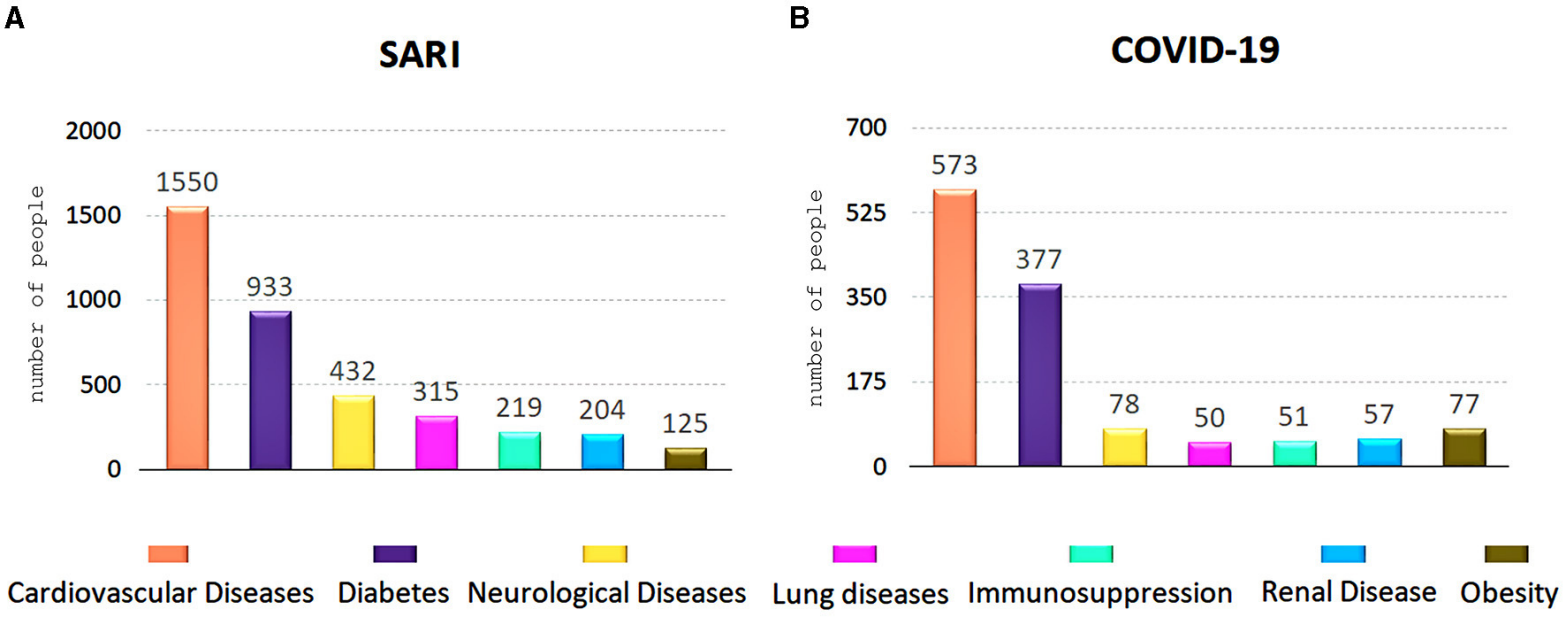
Underlying diseases present in 2,481 patients hospitalized for SARI **(A)** and for those 800 with COVID-19 diagnosis **(B)**.

The most common underlying illness are shown in Figure 3. Although we had information missing for a lot of cases, among 2,904 who had appropriately filled in these fields, 85% (*n* = 2,481) presented at least one comorbidity, and 33% (*n* = 830) were COVID-19 cases, i.e., 62% of the COVID-19 cases presented at least one comorbidity. Among all SARI inpatients, approximately 89% (*n* = 770) of non-survivors had some underlying disease. Multi-morbidities were present in 50% (*n* = 1,238) of SARI group, of which 32% (*n* = 400) were COVID-19 patients.

Risk factors associated with in-hospital death were evaluated with multivariate Poisson regression and multivariable Cox proportional hazards regression. In the total number of SARI cases, using both Poisson and Cox models, the following were predictors of mortality: older people, presence of neurological diseases, immunosuppression, obesity, and need for IVS. However, the Poisson test also revealed that the ICU admission (RR: 1.62; 1.33–1.98) and the COVID-19 diagnosis (RR: 1.24; 1.06–1.46) were also indicative factors of an increased risk for mortality, with female gender having a protective effect against death (RR: 0.85; 0.73–1.00) (Table 1). The use of Poisson distribution in this case (binary data with log link) enable to control the over/underdispersion to avoid the use of negative binomial distribution and robust estimation variance for binary outcome.

**Table 1:** Predictors for death and protective factors (both in bold) using both Poisson and Cox Models in SARI and COVID-19 subgroup.

	**Poisson**				**Cox**			
**Predictors**	**RR**	**CI (95%)**		***p-*** **values**	**HR**	**CI (95%)**		***p*** **-values**
**SARI**								
Older age	**1.03**	1.02	1.03	*0.000*	**1.03**	1.02	1.04	*0.000*
Neurological diseases	**1.47**	1.20	1.79	*0.000*	**1.43**	1.17	1.76	*0.001*
Immunossupression	**1.67**	1.30	2.15	*0.000*	**1.81**	1.40	2.33	*0.000*
Obesity	**1.46**	1.07	2.00	*0.018*	**1.34**	0.97	1.85	*0.076*
IVS	**2.29**	1.85	2.83	*0.000*	**1.77**	1.40	2.24	*0.000*
ICU admission	**1.62**	1.33	1.98	*0.000*	^**_**^	^**_**^	^**_**^	^_^
COVID-19	**1.24**	1.06	1.46	*0.008*	^**_**^	^**_**^	^**_**^	^_^
Female gender	**0.85**	0.73	0.99	*0.044*	^**_**^	^**_**^	^**_**^	^_^
**COVID-19**								
Older age	**1.02**	1.01	1.03	*0.000*	**1.03**	1.02	1.04	*0.000*
Neurological diseases	**1.67**	1.17	2.38	*0.004*	**1.41**	0.98	2.03	*0.063*
IVS	**1.81**	1.34	2.44	*0.000*	**1.84**	1.34	2.54	*0.000*
ICU Admission	**2.35**	1.76	3.14	*0.000*	**1.32**	0.97	1.80	*0.074*
Vaccination (doses)	^**_**^	^**_**^	^**_**^	^_^	**0.85**	0.74	0.99	*0.035*

RR, relative risks; HR, Hazard ratios; CI, confidence interval; ICU, intensive care unit; IVS, invasive ventilation support. The italic values is given only to differentiate the p-value from the other values.

In a sub-analysis for COVID-19, the predictors of mortality using both models were the following: older age, presence of neurological diseases, need for ICU and invasive ventilatory support. However, only the Cox model demonstrated that the higher number of vaccine doses was a protective factor for mortality (HR: 0.85; 0.74–0.99) (Table 1).

**Figure 4:**
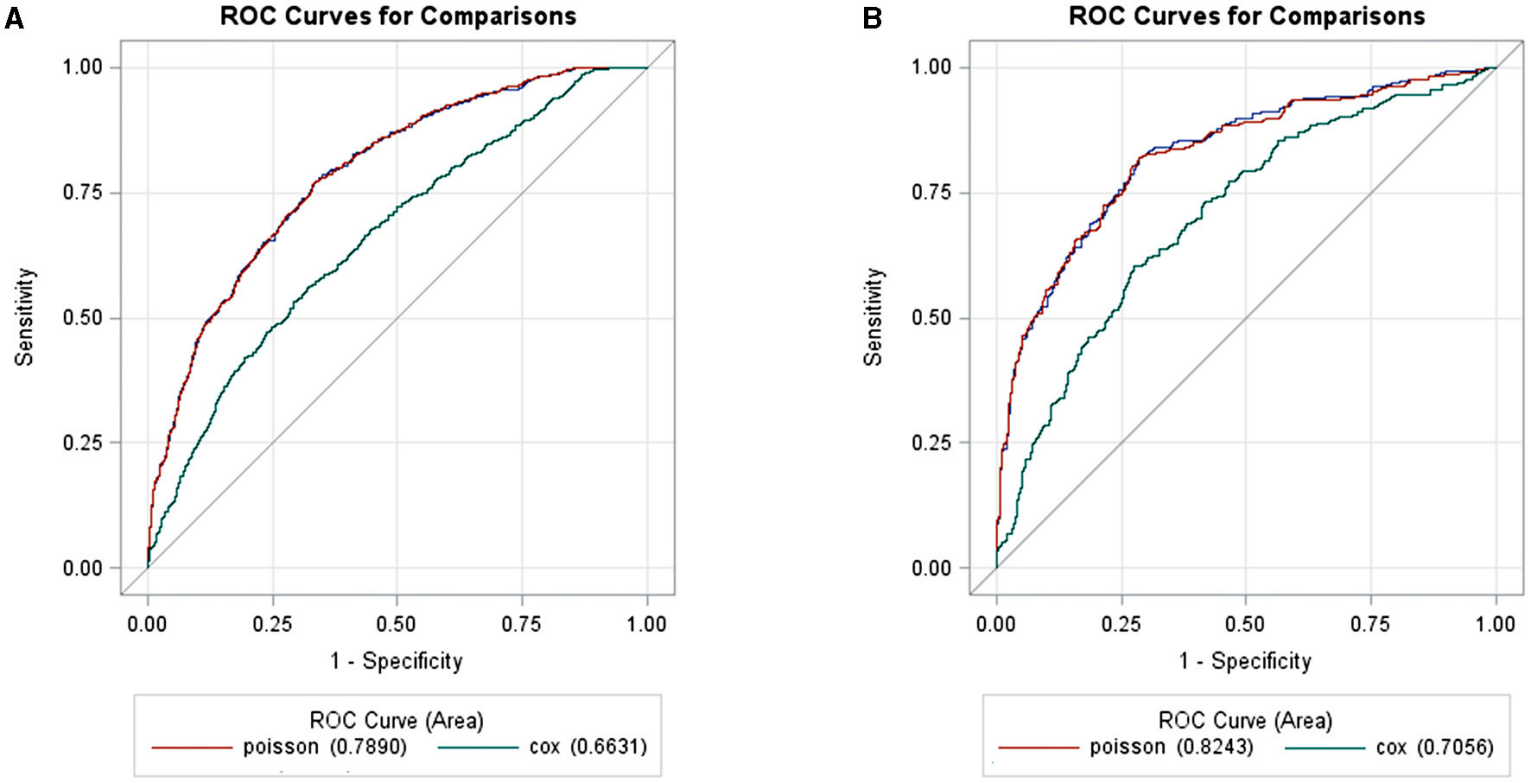
Two receiver operating characteristic curves (ROC) showed to compare the area under the curve (AUC), specificity and sensitivity values between Poisson and Cox models, for death outcome from predicted values obtained by each models. The comparison was made for SARI **(A)** and for COVID-19 **(B)** group. *p* < 0.0001 for both.

Additionally, considering the predicted values by each model and the calculation of a ROC curve, we showed that Poisson is the most suitable model for analyzing risk factors for death in patients with SARI/COVID-19 (Figure 4), due to its greater AUC when compared with Cox model (0.789 vs. 0.663, *p* < 0.0001), and its greater specificity (0.700 vs. 0.625) and sensitivity (0.750 vs. 0.625).

## Discussion

Historically, Brazil has had high adherence to previous vaccination campaigns. The mass vaccination campaign conducted in Botucatu City (on May 16 and Aug 08, 2021) was a success, allowing the immunization of 77,683 and 60,333 inhabitants (first and second dose, respectively) from a total of 92,394 adults (i.e., coverage 84 and 80%), in a record period. This resulted in a drastically reduced number of deaths especially in the pre-omicron period (11, 12).

Here we showed 1,338 (33%) hospital admissions for SARS-CoV-2 and 371 related deaths in Botucatu. This mortality rate of ~27% was similar to that found in other Brazilian studies (13–15). However, these data represent people of all ages, different variants distributed along the time, and a longer observation period (24 months) that included the months before the vaccines were made available, what is reinforced by the high frequency (69%) of unvaccinated people included here. It is well known that the benefits of vaccination are undeniable in protecting against severe COVID-19 (3, 12, 16), and its effects are even more protective according to the higher number of doses received and the younger age (3), especially due to factors related to immunosenescence (3, 17) reflecting in a lower vaccine response.

Regarding age, it is worth noting that about half of all hospitalizations for SARI and COVID-19 occurred in people older than 60 years in our study, which is consistent with the literature (2, 3, 17–19) and here, the mortality rate in this older adult population specifically by COVID-19 was 40%, higher than the rate found in younger people (16%). It shows us that COVID-19 was more aggressive than any other respiratory viruses in causing hospitalization, mainly in the older adult. Other studies have found higher mortality rate in those hospitalized for SARI due to COVID-19 than non-COVID-19 cases (20, 21). Among hospitalized inpatients from our study, 2,657 (66%) admissions and 495 (19%) deaths were non-COVID-19 related. The mortality rate in the older adult and younger overall (SARI) was ~- 20 and 22%, respectively. Independently of this, other respiratory viruses also have a high impact on public health (22), especially in the older population (17, 21) and policies to promote the prevention of SARI cases are, and certainly will continue to be necessary to entire population.

Several works reported a higher risk of a poor prognosis/death not only in older adult individuals, but also in those with comorbidities (4, 13, 14, 19, 20, 23, 24), not differing from the findings of the current study. At least one comorbidity was present in 85% (SARI group) and 62% (COVID-19 subgroup) of inpatients, and this last rate was similar to that found by Castro et al. (13). We observed the most common underlying illnesses were cardiovascular diseases and diabetes, followed by neurological disturbs, findings shared by Santos et al. (25) and Sousa et al. (19). Multi-morbidities were present in half cases of the SARI group and COVID-19 subgroup, and it is also a factor proven to be related to a higher risk/chance of death (4, 24).

The clinical manifestation of COVID-19, as well as some other viral conditions, is also highly heterogeneous and depends on the characteristics of the agent (variants) (26) and of the host. Among all hospitalized, low oxygen saturation (SpO2 < 95%), dyspnea, coughing, respiratory discomfort, and fever were the most expressive complaints, which converges with the literature (4, 18, 19). Since most of the hospitalized people in our study were from the first year of the pandemic, the most common symptoms found here, a combination of sore throat and head, were similar to those found by Prado et al. (4) in the same period. However, as the pandemic persisted and omicron became more prevalent lately, Sobral et al. (18) found respiratory discomfort and abdominal pain to be the most frequent manifestations.

As mentioned before, obtaining accurate estimates of the risk of COVID-19-related death in the population is challenging in the context of changing levels of circulating infection (27), types of circulating variants, the population heterogeneity, and regional and social factors (13, 16, 28–30). Besides, many studies which reported potential predictors of mortality in patients with COVID-19 with different methodological analyses were found in the literature. For this reason, we proposed a comparison between two statistical analyses to verify these predictors in all SARI inpatients.

Clinical prediction for mortality in patients with COVID-19 could help to identify those patients who require the most urgent help and make numerous medical decisions based on the risk of developing a particular outcome or state of health within a specific period. This also supports the efficient use of limited medical resources, reducing the impact on the healthcare system (29). The main prediction outcomes used in the studies are death, development of severe/critical state, ICU admission/mechanical ventilation/death, survival time, and length-of-hospital stay (7). Only the mortality outcome was investigated for its possible predictors in this study.

Our study showed three variables as predictors of mortality in both the SARI group and the COVID-19 subgroup, using Poisson and Cox: advanced age, SVI, and the presence of neurological disease. As mentioned above and according to the literature, the age is an important risk factor for a poor prognosis, not only for COVID-19, but also for any SARI case. These findings were from Brazilian studies using the Poisson (14, 20, 23, 28) and Cox models (4, 13, 15, 18, 24, 25). However, Sousa et al. (19) also considered both Poisson and Cox models, sharing very similar results-the older adult and people with comorbidities (CVD, neurological disease, lung disease) had a higher risk of dying from COVID-19. Furthermore, others (3, 21, 30) performed multivariate logistic regression for their analysis, and some results were similar. Although many studies use this last methodology, we decided not to consider it because when the event is not rare, the odds ratio (OR) can be overestimated (8).

The need for the use of IVS contributed to an increase in the probability of death, in this and in other studies, using Poisson (14, 20), Cox (15, 24) or logistic regression (30). As neurological diseases indicated high mortality, our study corroborates with several others, also using these statistical methodologies (19, 25). In addition to these Brazilian studies, the findings of the present study are supported by the systematic review and meta-analysis conducted by Shi et al. (2), in which the advanced age, male sex, preexisting comorbidities and complications during hospitalization are predictors of COVID-19 mortality.

Moreover, only in our SARI group other two preexisting medical conditions have predicted mortality: the presence of immunosuppression and obesity (by Poisson and Cox). Such underlying diseases have also been associated with a higher risk of death, especially in those COVID-19 cases, as reported in other studies assigning different analyses (Poisson, Cox and logistic regression) (19, 24, 31). Although we did not check whether multimorbidity would be a predictor of mortality, half of our inpatients had multimorbidities and in this way, Colnago et al. (3) by logistic regression, Mascarello et al. (23) by Poisson, Prado et al. (4) and Oliveira Lima et al. (24) by Cox previously demonstrated that fatality was higher among this population.

High fatality rates were observed among COVID patients admitted to the ICU admission using both Cox and Poisson. However, for SARI this predictor only appeared when using the Poisson model. Another variable that only appeared using Poisson, specifically for the SARI group, was COVID-19 infection as a mortality prediction and female gender as a protection factor. The literature (20) showed that ICU admission could be a predictor of mortality in people with respiratory infections, especially those positive for SARS-CoV-2. Regarding gender and disease progression, similarly to our results, Bermudi et al. (28) found that being male could be a predictor for death in these patients also using Poisson, and Colnago et al. (3), Castro et al. (13), Prado et al. (4) and Sobral et al. (18) pointed the same using other multivariate analyses.

We noted another difference using the two proposed models: only using Cox, the presence of anti-COVID-19 booster vaccine doses appeared as a protective factor against death. This data is aligned with that reported by Colnago et al. (3) during the Omicron wave in Brazil but using logistic regression. Jesus et al. (20) observed greater vulnerability, especially in the older adult who have received the inactivated virus vaccine suggesting the importance of giving the additional doses to this population as a priority.

However, it is worth remembering that this vaccine platform was applied to most of the older adult population in Brazil because it was the first vaccine to be approved by the local regulatory agency, so the age factor may also influence the lower protection conferred by it.

This discussion was based on Brazilian studies on predictors of mortality in cases of SARI, and it was noticeable that even having the same purpose, the studies use different types of multivariate analysis. We focused on showing mainly those works that used Poisson and Cox models here. Thus, the remaining question is: which analysis would be the most reliable, considering that the findings may be slightly different according to the model chosen? It is necessary a reflexive thought to answer this question.

The Poisson regression model is generally used in epidemiology to analyze longitudinal studies where the response is the number of episodes of an event occurring in a given time. The Cox regression model, in turn, is generally used to analyze the time to an event. Using both robust methods for variance estimation corrects the variance overestimation and produces adequate confidence intervals. The Cox and Poisson models also behaved well with the presence of continuous covariates (32). It is recognized that the time to outcome has two major advantages over cumulative outcome at a particular time: it is a more sensitive measure of efficacy and it also allows inclusion of individuals with unequal lengths of follow-up (8).

However, when we obtain relatively few results, the duration of follow-up is relatively short. Thus, fewer subjects are lost to follow-up using cumulative outcomes, and logistic regression will give similar results to using time-to-outcome and proportional hazards analysis. With Poisson regression, the outcome will be estimated to be zero or higher. In contrast to multiple linear regression the outcome can be estimated with negative values for certain subgroups of subjects-defined by independent variables–and it is clear that clinical events cannot have negative values (8). However, assuming that we are discussing respiratory infections with the rapid outcome, how important is it to slow down the progression of a disease? From our point of view, time is not a determining factor capable of changing the outcome; therefore, the outcome analyses can be carried out in a binary manner.

Although the notification of COVID-19 hospital admissions is compulsory in Brazil and the System used for the inclusion of participants in this study (SIVEP-gripe) provides the most representative account of SARI hospitalized patients in the entire city, whose data contained are more reliable than other COVID-19 surveillance systems (e.g., E-sus notifica), it must be considered that the use of the secondary database will always run into possible study biases. First, there may be a limitation in the predictors of the existing data with an impaired patient monitoring. Second, this dataset has a restricted number of variables (comorbidities, symptoms, medical procedures) with a lack of laboratory data, including for example, confirmatory tests for underlying diseases-that are only notified according to the patient's report or perception of the medical staff at the time of admission. Additionally, data entry with free-text fields causes an understandable discrepancy in the use of medical terms and descriptions, which leads to a lack of standardization in the completion of these data by health professionals. Other limitations include the lack of an adjustment for some subjects, clinical or regional characteristics (i.e., malnutrition, unhealthy health habits, treatments applied in the hospital or those chronically used by individuals, inequities and economic development to which the individual belongs, etc.).

## Conclusion

The findings of dichotomous and time-event predictor models may differ, and their significance depends on the epidemiological assumptions and on the research question. Considering the short time-course of SARI-COVID, the Poisson adjustment was more appropriate, since the occurrence or not of a certain outcome (death) in this case, is more crucial than considering when it occurred (early or late, and considering the proportional risks). In other words, choosing Poisson would simplify the model and provide precise results. Besides, in addition to identifying more associations using the Poisson model than Cox model, we demonstrated that Poisson was more sensitive and specific in the metric performance analysis comparing the both models.

In this manner, older age, neurological diseases, IVS and ICU admission were the four predictors of mortality to hospitalized COVID-19 patients, using Poisson regression. To the SARI group, in addition to the above predictors, others appeared in the Poisson model, such as obesity, immunosuppression and SARS-CoV-2 infection itself.

Lastly, the present study provides additional support for using other more suitable models as alternatives to logistic regression, available in most statistical design used for epidemiological studies analyses.

## Data availability statement

The raw data supporting the conclusions of this article will be made available by the authors, without undue reservation.

## Ethics statement

Research Ethics Committee of Botucatu Medical School (FMB/Unesp) approved the study (CAAE: 57919122.9.0000.5411). The studies were conducted in accordance with the local legislation and institutional requirements. The Ethics Committee/institutional review board waived the requirement of written informed consent for participation from the participants or the participants' legal guardians/next of kin because epidemiological study - secondary data.

## Author contributions

KT: Conceptualization, Data curation, Formal analysis, Investigation, Methodology, Project administration, Validation, Writing—original draft. CA: Investigation, Visualization, Writing—review & editing. RG: Investigation, Validation, Writing—review & editing. LdM: Investigation, Validation, Writing—review & editing. PA: Investigation, Validation, Writing—review & editing. CF: Conceptualization, Data curation, Formal analysis, Investigation, Methodology, Supervision, Validation, Writing—review & editing, Writing—original draft.
