# Supplementary material for: Dichotomous outcomes vs. survival regression models for identification of predictors of mortality among patients with severe acute respiratory illness during COVID-19 pandemics
Source: Front Public Health. 2023 Dec 6;11:1271177. doi: 10.3389/fpubh.2023.1271177 (PMC10732580; doi:10.3389/fpubh.2023.1271177)
Supplement: Supplementary file 2 [file Table_1.DOCX]

**Suppl / Table** – Immunization of patients hospitalized with COVID-19 in Botucatu/SP from March 2020 to March 2022, according to gender.


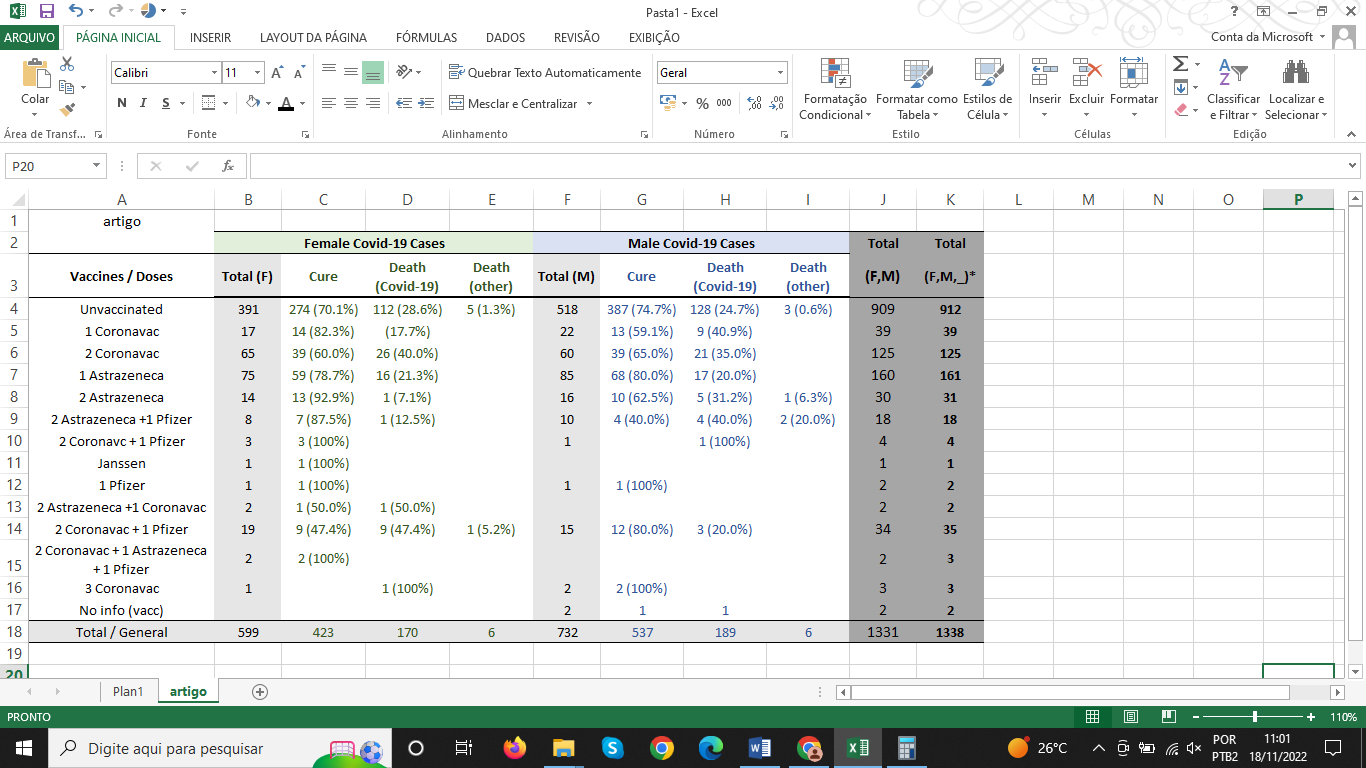


* data referring to the total number of each category, considering female, male and those who did not present such information in the database.
